# Learning Observer-Based Sensor Fault-Tolerant Control of Distributed Generation in an Islanded Microgrid for Bus Voltage Stability Enhancement

**DOI:** 10.3390/s22186907

**Published:** 2022-09-13

**Authors:** Jingfeng Mao, Chunyun Yin, Xiaotong Zhang, Aihua Wu, Xudong Zhang

**Affiliations:** School of Electrical Engineering, Nantong University, Nantong 226019, China

**Keywords:** distributed generation, islanded microgrid, fault-tolerant control, sensor fault, learning observer, feedback control

## Abstract

In order to improve robust operating performance and enhance bus voltage stability, a learning observer-based fault-tolerant control strategy is proposed for the distributed generation in islanded microgrid with sensor faults and uncertain disturbances. Firstly, the output feedback control theory and the linear matrix inequality method are used to design closed-loop controller for the voltage source inverter of distributed generation; secondly, a fault-tolerant model and control structure of the distributed generation in an islanded microgrid with sensor faults is analyzed. By employing the fault output signal conversion filter and proportional derivative type learning observer, the online estimation and real-time compensation of the sensor fault signal are realized. Thirdly, the system synthesis of output feedback control and fault-tolerant control is completed. Finally, the multi-scenario sensor fault scheme simulation experiment verifies that the proposed control strategy has strong sensor fault tolerance and adaptability.

## 1. Introduction

In recent years, due to the shortage of fossil energy and the crisis of the global ecological environment, distributed generation (DG) technologies based on renewable energy applications have undergone rapid development. As an effective operational organization form of DG, a microgrid can run in grid-connected mode or in islanded mode. When the microgrid runs in the islanded mode, there are still many challenges and problems in bus voltage stability control and reliable operation due to the lack of power support from the large grid [1,2,3,4].

In islanded mode, the microgrid bus voltage and frequency must be regulated only by the DG. In particular, the inverter of DG plays an important role as a power exchange interface with the microgrid. For example, reference [5] proposed a voltage–power control method to improve the parallel operation performance of DG based on the voltage source inverter (VSI). Reference [6] proposed an accurate proportional power sharing method for a VSI-based islanded microgrid with minimal communication requirements. Reference [7] proposed an adaptive sliding mode controller to improve the anti-disturbance ability of the islanded parallel VSI control system and ensure the global robustness of the bus voltage stabilization. In refs. [8,9], multivariable closed-loop feedback control methods are used to design the VSI voltage controllers through a *dq* frame for the islanded mode. No matter what control method is used, it is necessary to accurately detect the DG output voltage and current. These detection results are directly used in the control algorithm operation of the inverter. Therefore, the accuracy of detecting voltage and current is crucial to the reliable operation of the DG in islanded microgrid.

Sensors are one of the most important and frequently faulty components in a detection system. If the voltage sensor and current sensor used to detect the DG output signal are faulty, the control output signal of the closed-loop controller will be wrong, causing a series of reactions, such as the unreliable output of the VSI, and even leading to the unstable operation of the microgrid [10,11,12].

Therefore, it is particularly important to carry out research on the fault-tolerant control of the microgrid under sensor fault conditions [13,14]. However, there are few relevant scenario studies at present, and only some references focus on the design of a power conversion controller based on the fault-tolerant control technology of fault observation and reconstruction. Reference [15] proposed a sensor fault observer for 5 KW wind power conversion system based on an estimation/decoupling strategy. In this method, the sensor fault is regarded as an unknown input variable. The system state variables and disturbance variables are estimated by the extended state observer (ESO), and a T-S fuzzy unknown input observation model is introduced to realize the identification and control compensation of sensor faults. Reference [16] proposed a Luenberger state observer (LSO) to estimate the faults of current sensors and DC link voltage sensors. Based on the detection of a possible sensor failure by the LSO, a reconstruction of the PWM rectifier control system is implemented to ensure reliable operation. Reference [17] studied the fault-tolerant attitude stabilization control of satellite systems under the conditions of actuator failure, sensor failure and external disturbance. Aiming at the faults of actuators and sensors in satellite systems, an output feedback fault-tolerant control scheme based on H_∞_ observer was proposed. The authors in reference [18] investigated the sensor fault diagnostics and fault-tolerant control for a class of microgrid based on voltage source converter by using the sliding-mode observer. The sensor faults can be reconstructed through the sliding-mode observer and transferred to the control block. Then the simulation results were given on the basis of the estimation and estimated errors of states/output, the reconstruction of faults, the estimation of disturbances, and the control performance of fault-tolerance. However, the designs of the above observers are relatively complex and some preconditions must be given, which has a certain impact on the robustness of fault estimation.

In recent years, the observer-based method was widely used for its simple structure and easy to design. In considerable research, such as references [19,20], the authors investigated the compensation method for the actuator and attitude sensor faults of satellite based on the PD-type learning observers. A fault tolerant control strategy was studied in reference [21] based on the PD-type learning observers to control the rigid spacecraft attitude systems with external disturbances, parameter uncertainties and actuator faults. The authors in reference [22] put forward an observer-based adaptive learning control strategy to stabilize the attitude of the spacecraft affected by the actuator failure. Then, a compound control law was proposed by combining the above observer and robust feedback controller for the stability of the spacecraft attitude. However, there are few results on the estimation of sensor faults based on PD-type learning observer for the microgrid applications, which is the motivation of this paper.

In this paper, an observer-based bus voltage stability fault-tolerant control strategy is proposed for the robust operation of DG in islanded microgrid with sensor failure. The control scheme adopts output feedback control theory and proportional derivative (PD) type learning observer fault diagnosis theory. The PD-type learning observer is designed to estimate and compensate for sensor faults in the output voltage and current *dq* components of the DG in islanded microgrid. The estimated faults are used for control reconstruction to ensure stable and fault-tolerant operation of the DG in the islanded microgrid.

Briefly, the main contributions of this paper are: (1) A mathematical model of the DG in islanded microgrid with sensor faults and uncertain disturbances is built. (2) A microgrid DG fault-tolerant controller is designed by synthesizing a PD-type learning observer and output feedback controller. (3) To compare the performance of the proposed control with previous existing methods, a PID controller are conducted. (4) Multi-scenario sensor fault scheme simulation experiments verify the correctness and effectiveness of the proposed fault-tolerant control strategy.

The rest of this paper is organized as follows. In Section 1, the structure of the DG in islanded microgrid is presented. Section 2 analyzes the voltage closed-loop output feedback control system and fault model with sensor failure. Section 3 introduces the design process of fault-tolerant control strategy. Section 4 presents the system simulation results and analysis to evaluate the effectiveness of the proposed fault-tolerant control strategy. Finally, Section 5 draws conclusions.

## 2. DG Structure and Mathematical Model in Islanded Microgrid

**Figure 1:**
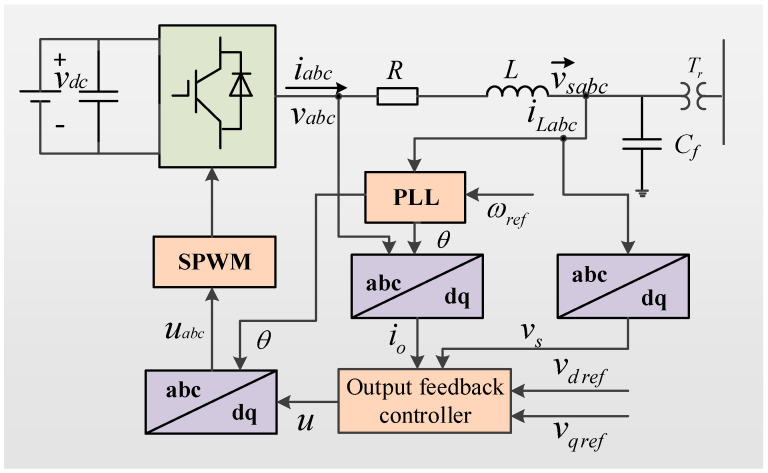
Schematic diagram of the DG structure in islanded microgrid.

Figure 1 is a schematic diagram of the structure of an islanded microgrid with one DG. As can be seen in Figure 1, the input voltage *v_dc_* of DG is converted by a voltage source inverter, and the inverter output voltage *v_abc_* is connected to the AC grid through the *LC* filter.

In Figure 1, the inverter adopts a six-pulse bridge power circuit structure; *L* and *C_f_* are the inductance and capacitance of the *LC* filter, respectively; *R* is the total equivalent loss resistance of the inverter; *θ* is the phase angle of the inverter output voltage; and PLL is the phase-locked loop.

According to Figure 1, the electrical dynamic Equations of the DG in the *abc* coordinate system can be expressed as [23]:

(1) $$\left\{ \begin{array}{l} L\frac{di_{abc}(t)}{dt}=-Ri_{abc}(t)+v_{abc}(t)-v_{sabc}(t)\\ C_{f}\frac{dv_{sabc}(t)}{dt}=i_{abc}(t)-i_{Labc}(t) \end{array}\right.$$

where *v_abc_*(*t*) and *i_abc_*(*t*) are the inverter output voltage and current, respectively, and *v_sabc_*(*t*) and *i_Labc_*(*t*) are the voltage and current of the DG connected to the island microgrid, respectively.

Assuming that the DC side input voltage of the DG is constant, for a three-phase inverter control system with SPWM modulation method, the per-cycle average value of the AC side voltage is related to the DC side input voltage as follows:

(2) $$v_{abc}(t)=\frac{v_{dc}}{2}m_{abc}(t)$$

where *m_abc_*(*t*) is the modulation signal of PWM method, which is used to control the inverter output voltage.

In the *dq* coordinate system, Equation (1) can be expressed as:

(3) $$\begin{array}{l} {\dot{i}}_{d}=-\frac{R}{L_{1}}i_{d}+\omega i_{q}-\frac{v_{sd}}{L_{1}}+\frac{v_{dc}}{2L_{1}}m_{d}\\ {\dot{i}}_{q}=-\omega i_{d}-\frac{R}{L_{1}}i_{q}-\frac{v_{sq}}{L_{1}}+\frac{v_{dc}}{2L_{1}}m_{q}\\ {\dot{v}}_{sd}=\frac{1}{C_{f}}\left(i_{d}-i_{Ld}\right)+\omega v_{sd}\\ {\dot{v}}_{sq}=\frac{1}{C_{f}}\left(i_{q}-i_{Lq}\right)-\omega v_{sq} \end{array}$$

where the subscripts *d* and *q* represent the *d*-axis and *q*-axis components; *ω* is the angular frequency, *ω* = 2*πf*, *f* is the frequency of the VSI output voltage and current; and *m_d_* and *m_q_* represent pulse width modulation signal in the *dq* coordinate.

Applying the Park transform operation to Equation (2), the following can be obtained:

(4) $$v_{d}=\frac{v_{dc}}{2}m_{d},v_{q}=\frac{v_{dc}}{2}m_{q}$$

where *v_d_* and *v_q_* are the inverter output voltage components in the *dq* coordinate system, respectively.

According to Equation (3), the DG mathematical model can be simplified as a state space form:

(5) $$\begin{array}{l} \dot{x}(t)=Ax(t)+Bu(t)+Hz(t)\\ y(t)=Cx(t) \end{array}$$

where *x* is the state variable, *x* = [*i_d_ i_q_ v_sd_ v_sq_*]^T^; *y* is the system output variable; *C* = *I*_4_ is the unit vector; *u* is the control input, *u* = [*m_d_ m_q_*]^T^; *z* is the disturbance term, *z* = [*i_Ld_ i_Lq_*]^T^; and *i_Ld_* and *i_Lq_* are the components of the DG output current in the *d*-axis and *q*-axis respectively. Respectively, the state matrices *A*, *B*, *H* can be expressed as:

$$A=\left[\begin{array}{cccc} -\frac{R}{L_{1}} & \omega & -\frac{1}{L_{1}} & 0\\ \omega & -\frac{R}{L_{1}} & 0 & -\frac{1}{L_{1}}\\ \frac{1}{C_{f}} & 0 & 0 & \omega \\ 0 & \frac{1}{C_{f}} & -\omega & 0 \end{array}\right],B=\frac{v_{dc}}{2}\left[\begin{array}{ll} \frac{1}{L_{1}} & 0\\ 0 & \frac{1}{L_{1}}\\ 0 & 0\\ 0 & 0 \end{array}\right],H=\left[\begin{array}{ll} 0 & 0\\ 0 & 0\\ \frac{-1}{C_{f}} & 0\\ 0 & \frac{-1}{C_{f}} \end{array}\right]$$

## 3. DG Closed-Loop Control and Fault Model

### 3.1. Fault Analysis and Controller Design

Accurate voltage and current detections are essential to ensuring the reliable operation of the DG in islanded microgrid, as these detections are used to calculate the PWM modulation signal that drives the inverter. Any errors in these detections could seriously disturb the operation of the DG in an islanded microgrid. Since the microgrid operates in islanded mode without the support of the main grid, detection errors can also adversely affect the bus voltage of the microgrid. Incorrect voltage and current detection s are mainly attributable to the failure of related detection equipment, such as sensor output error failure, sensor accuracy failure, sensor signal loss, etc.

Therefore, this paper carries out fault-tolerant control for the DG in islanded microgrid with sensor (current sensor or voltage sensor) faults, so as to improve the operating robustness performance of the DG and enhance the bus voltage stability of the microgrid.

**Figure 2:**
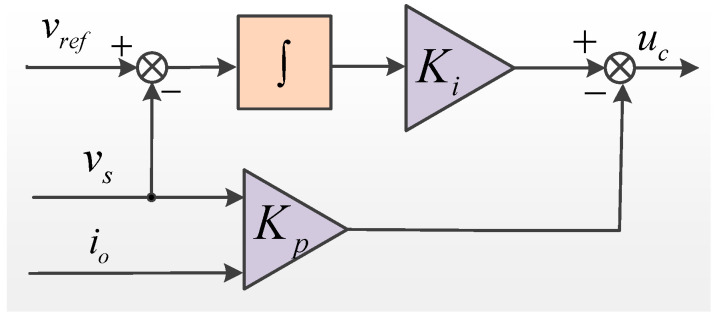
Output feedback controller.

When the sensor works normally, the voltage closed-loop output feedback controller of DG can be designed as shown in Figure 2.

The output feedback control law is defined as [24]:

(6) $$u_{c}=K_{i}\int \left(v_{ref}-v_{s}\right)-K_{p}y_{s}$$

where *y_s_* = [*i_o_ v_s_*] is the DG actual output variable when the sensor operates normally, *v_s_* = [*v_sd_ v_sq_*]^T^, *i_o_* = [*i_d_ i_q_*]^T^; *v_ref_* is the reference input voltage vector in the *dq* coordinate system, *v_ref_* = [*v_sdr_ v_sqr_*]^T^; and *K_p_* and *K_i_* are the control law proportional gain matrix and integral gain matrix, respectively, letting *K* = [−*K_p_* *K_i_*]^T^.

In Figure 2, the reference input voltage vector *v_ref_* and the actual measured output voltage vector *v_s_* are adopted to the output feedback controller as to calculate and generate the control signal *u_c_*.

In order to make the DG in the islanded microgrid operate more stably, an augmented system model with an output feedback controller is considered because of the external disturbance. It is expressed as:

(7) $$\begin{array}{l} {\dot{x}}_{a}\left(t\right)=A_{a}x_{a}\left(t\right)+B_{a}u_{c}\left(t\right)+H_{a}\tilde{z}\left(t\right)\\ y_{a}(t)=Cx_{a}(t) \end{array}$$

where *x_a_* = [*x*^T^ *η*^T^]^T^, *y_a_* is the system output variable with an output feedback controller and $\eta =\int \left(v_{ref}-v_{s}\right)$ is the output of the integral term of the output feedback controller; each matrix is defined as:

$$\begin{array}{c} A_{a}=\left[\begin{array}{cc} A & 0\\ -C_{i} & 0 \end{array}\right],B_{a}=\left[\begin{array}{c} B\\ 0 \end{array}\right]\\ H_{a}=\left[\begin{array}{cc} H & 0\\ 0 & I \end{array}\right],C_{i}=\left[\begin{array}{llll} 0 & 0 & 1 & 0\\ 0 & 0 & 0 & 1 \end{array}\right],\tilde{z}=\left[\begin{array}{c} z\\ v_{ref} \end{array}\right] \end{array}$$

Here, the linear matrix inequality LMI method is used to determine the control law gain matrix *K* [25,26], assuming that there is a positive definite matrix *M* > 0 satisfying the following inequality:

(8) $$\left[\begin{array}{ccc} \left(\partial M+M\partial^{T}\right) & H_{a} & M^{T}\\ H_{a}{}^{T} & -I & 0\\ M & 0 & -\beta^{2}I \end{array}\right]<0$$

where ∂ = *A_a_* + *B_a_K*.

Through the LMI minimization method, the corresponding matrices *M* and *K* can be solved, under the condition that the value of the linear matrix inequality variable *β* is minimized. The controller designed by the above-mentioned minimization method can effectively suppress the influence of disturbance on the system, thereby enhancing the robustness of the controller and ensuring the realization of the control objective.

In order to determine the controller gain matrix *K* = [−*K_p_* *K_i_*]^T^, define Ω = *KM* and rewrite the above inequality as an LMI:

(9) $$\left[\begin{array}{ccc} \left(A_{a}M+MA_{a}{}^{T}+B_{a}\Omega +\Omega^{T}B_{a}{}^{T}\right) & H_{a} & M^{T}\\ H_{a}{}^{T} & -I & 0\\ M & 0 & -\beta^{2}I \end{array}\right]<0$$

Finally, Ω and *M* are obtained by programming, and the gain matrix *K* is determined as follows:

*K* = Ω*M*^−1^ (10)

When the sensor works in a fault state, the proposed fault-tolerant control law *u* can be designed as follows:

(11) $$u=u_{c}+u_{f}$$

where *u_f_* is the output feedback control law with fault compensation, which will be designed in the next section.

### 3.2. System Model with Sensor Faults

For the DG in the islanded microgrid, a system model considering sensor faults can be established as:

(12) $$\begin{array}{l} \dot{x}(t)=Ax(t)+Bu(t)+Hz(t)\\ y_{b}(t)=Cx(t)+D_{s}f_{s}(t) \end{array}$$

where *y_b_* is the system output variable considering sensor faults; *D_s_* = *I*_4_ is the error vector coefficient matrix, which represents the selection of the fault signal; and *f_s_* represents the fault signal of the output current and voltage of the DG in islanded microgrid, which can be expressed as:

(13) $$f_{s}={\left[\begin{array}{cc} f_{i}{}^{T} & f_{v}{}^{T} \end{array}\right]}^{T}$$

where *f_i_* = [Δ*_id_* Δ*_id_*]^T^ is the current detection error, and *f_v_* = [Δ*_vd_* Δ*_vd_*]^T^ is the voltage detection error.

## 4. Fault-Tolerant Control Based on PD-Type Learning Observer

In this paper, the fault diagnosis and estimation method of the PD-type learning observer is used to realize quickly and accurately estimate and compensate the sensor faults in the operation of DG in an islanded microgrid.

### 4.1. Design of PD-Type Learning Observer

For the DG model Equation (12), in an islanded microgrid, considering sensor faults, the fault conversion filter can be designed as:

(14) $${\dot{x}}_{s}(t)=-A_{s}x_{s}(t)+A_{s}y(t)$$

where *x_s_* is the state variable of the filter that represents the filter system detection, and *A*_s_ is the Hurwitz matrix.

Combining Equations (12) and (14), and letting *x*(*t*) = [*x^T^*(*t*) *x_s_^T^*(*t*)]^T^, the system augmented fault model can be established as:

(15) $$\begin{array}{l} \dot{\bar{x}}(t)=\bar{A}\bar{x}(t)+\bar{B}u(t)+\bar{H}z(t)+{\bar{D}}_{s}f_{s}(t)\\ {\bar{y}}_{s}(t)=\bar{C}\bar{x}(t) \end{array}$$

where ${\bar{y}}_{s}$ is the output of the DG in an islanded microgrid, considering the conversion filter, $\bar{A}$, $\bar{B}$, $\bar{C}$, $\bar{H}$ and ${\bar{D}}_{S}$ are the system state matrices, respectively, which are defined as:

$$\bar{A}=\left[\begin{array}{cc} A & 0\\ A_{s} & -A_{s} \end{array}\right],\bar{B}=\left[\begin{array}{c} B\\ 0 \end{array}\right]$$

$$\bar{H}=\left[\begin{array}{c} H\\ 0 \end{array}\right],{\bar{D}}_{s}=\left[\begin{array}{c} 0\\ A_{s} \end{array}\right],\bar{C}=\left[\begin{array}{cc} 0 & I_{4} \end{array}\right]$$

The dimension of input variables, output and fault vectors in the system augmented fault model (15) are the same as that in the original system model (12).

From the model Equation (15), it can be seen that the original sensor fault issue has been transformed into the actuator fault issue.

For the augmented fault model (13), a PD-type learning observer is designed as:

(16) $$\begin{array}{l} \dot{\hat{\bar{x}}}(t)=\bar{A}\hat{\bar{x}}(t)+\bar{B}u(t)+{\bar{D}}_{s}{\hat{f}}_{s}(t)+T{\bar{e}}_{y}(t)\\ {\hat{\bar{y}}}_{s}(t)=\bar{C}\hat{\bar{x}}(t)\\ {\bar{e}}_{y}(t)=\bar{y}(t)-{\hat{\bar{y}}}_{s}(t)\\ {\hat{f}}_{s}(t)={\hat{f}}_{s}(t-\tau )+Q\left(\sigma {\bar{e}}_{y}(t)+{\dot{\bar{e}}}_{y}(t-\tau )\right) \end{array}$$

where ${\bar{e}}_{y}$ is the output error and ${\dot{\bar{e}}}_{y}$ is the integral term of the output error; $\hat{\bar{x}}(t)$, ${\hat{\bar{y}}}_{s}(t)$ and $\hat{f}(t)$ are the system state estimation, detection output estimation and sensor fault compensation signals of the observer, respectively; *T* and *Q* are the learning observer matrix, *σ* is the constant to be determined; and *τ* is the learning time interval.

Respectively, define the control system state estimation error $e_{\bar{x}}$, the output estimation error $e_{{\bar{y}}_{s}}$ and the actuator fault reconstruction error $e_{f_{s}}$ as:

(17) $$\begin{array}{l} e_{\bar{x}}(t)=\bar{x}(t)-\hat{\bar{x}}(t)\\ e_{{\bar{y}}_{s}}(t)={\bar{y}}_{s}(t)-{\hat{\bar{y}}}_{s}(t)\\ e_{f_{s}}(t)=f_{s}(t)-{\hat{f}}_{s}(t) \end{array}$$

From the augmented fault model Equation (15) and the PD-type learning observer Equation (16), the estimation error dynamic Equation can be expressed as:

(18) $$\begin{array}{l} {\dot{e}}_{\bar{x}}(t)=\left(\bar{A}-T\bar{C}\right)e_{\bar{x}}(t)+{\bar{D}}_{s}\left(f_{s}(t)-{\hat{f}}_{s}(t)\right)\\ e_{{\bar{y}}_{s}}(t)=\bar{C}e_{\bar{x}}(t) \end{array}$$

### 4.2. Observer Stability Analysis

The designed PD-type learning observer can estimate the sensor faults when the condition ||${\bar{f}}_{s}$(*t*)||_∞_ ≤ *n* is satisfied.

Where

(19) $${\bar{f}}_{s}(t)=f_{s}(t)-f_{s}(t-\tau )$$

In order to estimate the sensor fault accurately and quickly, the following theorem is obtained.

Theorem: Assuming that the sensor fault satisfies the above conditions, if a positive scalar *σ* ≥ 1, positive definite symmetric matrices *P* and *G*, and matrices *Z* and *Q* exist, then the following inequality holds.

(20) $$P\bar{A}+{\bar{A}}^{T}P-Z\bar{C}-{\bar{C}}^{T}Z^{T}+G<0$$

(21) $$\left[\begin{array}{cc} -G & ({\bar{A}}^{T}P-{\bar{C}}^{T}Z^{T}){\bar{D}}_{s}\\ W & -I_{2} \end{array}\right]<0$$

(22) $${\bar{D}}_{s}{}^{T}P=Q\bar{C}$$

(23) $$K\bar{C}{\bar{D}}_{s}-I_{3}=0$$

where *Z* = *PT*, and *W* is symmetric matrix term.

The proposed PD-type learning observer can guarantee that the augmented state estimation error and fault estimation error are ultimately uniformly bounded.

Proof: Choose the Lyapunov function as:

(24) $$V(t)=e_{\bar{x}}{}^{T}(t)Pe_{\bar{x}}(t)+\int_{t-\tau }^{t}e_{\bar{x}}{}^{T}(s)Ge_{\bar{x}}(s)ds$$

By deriving the above Lyapunov function and letting *J* = $\bar{A}-T\bar{C}$, one obtains:

(25) $$\begin{array}{ll} \dot{V}(t)= & e_{\bar{x}}{}^{T}(t)\left[PJ+J^{T}P+G\right]e_{\bar{x}}(t)-\\ & e_{\bar{x}}{}^{T}(t-\tau )Ge_{\bar{x}}(t-\tau )+\\ & 2e_{\bar{x}}{}^{T}(t)P{\bar{D}}_{s}f_{s}(t)-\\ & 2e_{\bar{x}}{}^{T}(t)P{\bar{D}}_{s}{\hat{f}}_{s}(t) \end{array}$$

Taking the fault compensation signal in Equation (17) into Equation (25) and employing Equation (22) results in:

(26) $$\begin{array}{ll} \dot{V}(t)= & e_{\bar{x}}{}^{T}(t)\left(PJ+J^{T}P+G-2\sigma P{\bar{D}}_{s}Q\bar{C}\right)e_{\bar{x}}(t)-\\ & e_{\bar{x}}{}^{T}(t-\tau )Ge_{\bar{x}}(t-\tau )+2e_{\bar{x}}{}^{T}(t)P{\bar{D}}_{s}{\hat{\bar{f}}}_{s}(t)-\\ & 2e_{\bar{x}}{}^{T}(t)P{\bar{D}}_{s}Q\bar{C}Je_{\bar{x}}(t-\tau )\\ & \leq e_{\bar{x}}{}^{T}(t)(PJ+J^{T}P+G-2\sigma P{\bar{D}}_{s}Q\bar{C}+\\ & 2P{\bar{D}}_{s}{\bar{D}}_{s}{}^{T}P)e_{\bar{x}}(t)-e_{\bar{x}}{}^{T}(t-\tau )Ge_{\bar{x}}(t-\tau )+\\ & e_{\bar{x}}{}^{T}(t-\tau )\left[{\left(Q\bar{C}J\right)}^{T}Q\bar{C}J-G\right]e_{\bar{x}}(t-\tau )+\\ & {\hat{\bar{f}}}_{s}{}^{T}(t){\hat{\bar{f}}}_{s}(t) \end{array}$$

Selecting the positive scalar *σ* ≥ 1, and taking Equation (20) into the result, one obtains:

(27) $$\begin{array}{ll} \dot{V}(t)\leq & e_{\bar{x}}{}^{T}(t)\left(PJ+J^{T}P+G\right)e_{\bar{x}}(t)+\\ & e_{\bar{x}}{}^{T}(t-\tau )\left[{\left({\bar{D}}_{s}{}^{T}PJ\right)}^{T}{\bar{D}}_{s}{}^{T}PJ-G\right]e_{\bar{x}}(t-\tau )\\ & +{\hat{\bar{f}}}_{s}{}^{T}(t){\hat{\bar{f}}}_{s}(t) \end{array}$$

Letting $\chi =P\bar{A}+{\bar{A}}^{T}P-Z\bar{C}-{\bar{C}}^{T}Z^{T}+G$, Equation (26) can be simplified as:

(28) $$\dot{V}(t)\leq -\xi {\left\|e_{\bar{x}}(t)\right\|}_{2}^{2}+n^{2}\leq 0$$

where $\xi =\lambda_{min}\left(-\chi \right)$.

According to the Lyapunov stability theory, the system state estimation error $e_{\bar{x}}$ and the fault estimation error $e_{f_{s}}$ are eventually uniformly bounded. The designed observer is reasonable, which can accurately estimate the sensor fault.

### 4.3. Synthesis of Fault-Tolerant Control System

In order to design the observer matrix *T* to satisfy Equations (20)–(22), the issue of solving Equation (22) is transformed into the following inequality issue [27]:

(29) $$\left[\begin{array}{cc} -\gamma I_{3} & {\bar{D}}_{s}{}^{T}P-Q\bar{C}\\ W & -I_{8} \end{array}\right]<0$$

Thus, the issue of solving observer *T* is transformed into the issue of finding the minimum value of Equations (20), (21) and (29). By selecting a sufficiently small scalar *γ* and using LMI toolbox in MATLAB to solve three inequalities, the values of matrix *P*, *Z* and *Q* can be obtained, and then the value of matrix *T* can also be obtained.

**Figure 3:**
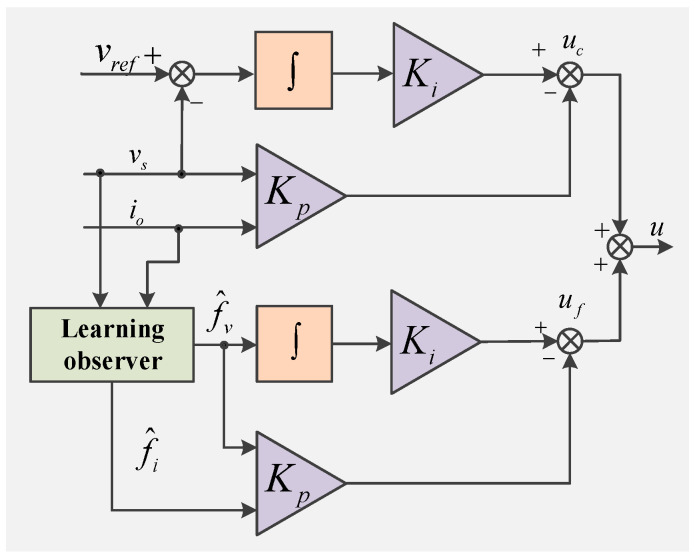
Fault-tolerant controller.

Figure 3 is a schematic diagram of the learning observer-based fault-tolerant control of the DG in the islanded microgrid with sensor faults.

According to Figure 3, the purpose of fault-tolerant control is to ensure that the DG in an islanded microgrid achieves voltage stability operation both with and without sensor faults conditions. The fault-tolerant control law *u* includes the normal control law *u_c_* under the working condition of the sensor without fault and the fault compensation control law *u_f_* under the working conditions of the sensor failure.

The fault compensation control law *u_f_* can be designed as:

(30) $$u_{f}=K_{i}\int {\hat{f}}_{v}-K_{p}{\hat{f}}_{s}$$

where ${\hat{f}}_{s}=\left[\begin{array}{cc} {\hat{f}}_{i} & {\hat{f}}_{v} \end{array}\right]$ is the estimated value of the fault.

Combining Equations (5) and (30), the final fault-tolerant control law *u* is obtained as [28]:

(31) $$\begin{array}{ll} u & =u_{c}+u_{f}\\ & =K_{i}\int \left(v_{ref}-v\right)-K_{p}y \end{array}$$

where $v=v_{s}-{\hat{f}}_{v}$, *v* is the output voltage with fault compensation.

According to the previous theoretical analysis, the advantages of the PD-type learning observer are summarized as follows: (1) There are few parameters and simple structure of the PD-type learning observer and (2) the computational complexity of the PD-type learning observer is greatly reduced. In the process of reconstructing the fault, the designer only calculates the historical information of faults and the estimation errors of the detection outputs without the integral ones; (3) the arbitrary error in sensors can be estimated by the PD-type learning observer; (4) the constraint that the control input is bounded can be relaxed under the PD-type learning observers; (5) the gain matrices of PD-type of observer can be solved by LMIs instead of haphazard selection; and (6) combining the PD-type observer with output feedback controller, the robustness of the DG operation can be guaranteed better and the stability of microgrid bus voltage can be improved.

## 5. System Simulation and Analysis

In order to verify the effectiveness of the fault-tolerant control strategy proposed in this paper, simulation analysis for multiple scenario sensor fault conditions was carried out.

### 5.1. System Parameter Setting

Aiming at the islanded microgrid circuit structure shown in Figure 1, a system simulation model was established using the MATLAB software platform. The DG circuit parameters are shown in Table 1.

According to the design method in Section 3.1, by constructing the augmented system model and using the LMI toolbox in MATLAB software, the parameters of the output feedback controller are obtained as:

$$K_{p}=\left[\begin{array}{cc} 0.35 & 0.03\\ -0.03 & 0.35 \end{array}\right],K_{i}=\left[\begin{array}{cc} 21.3 & 0\\ 0 & 21.3 \end{array}\right]$$

The learning observer matrix parameters can be calculated by the design method described in Section 4.3. The scalar *γ* is set to 10^−6^, *σ* is set to 1 and the time interval *τ* is set to 0.005 s. Using the LMI minimization approach to optimize the matrix parameters and solve the minimum value of inequality, the solution of matrix *Q* is obtained as:

$$Q=\left[\begin{array}{cccccc} 0 & 0 & 0 & 22.54 & 0 & 0\\ 0 & 0 & 0 & 0 & 24.73 & 0\\ 0 & 0 & 0 & 0 & 0 & 25.63 \end{array}\right]$$

Further, the solution of matrix *T* is obtained as:

$$T=\left[\begin{array}{cccccc} 2.48 & 0 & 0 & 1.15 & 0 & 0\\ 0 & 2.56 & 0 & 0 & 1.08 & 0\\ 0 & 0 & 2.39 & 0 & 0 & 1.15\\ 0.23 & 0 & 0.04 & 2.63 & 0 & 0.01\\ 0 & 0.028 & 0 & 0 & 2.87 & 0\\ -0.04 & 0 & 0.031 & -0.01 & 0 & 2.65 \end{array}\right]$$

To compare the performance of the proposed control with previous existing methods, a PID controller was conducted. The PID control law parameters, *K_cp_*, *K_ci_* and *K_cd_*, were selected by using the classical experimental optimization method, which is a common method for highly nonlinear and uncertain control system design. To ensure the PID closed-loop system has good response characteristics, the control parameters are determined as follows: *K_cp_* = 0.7, *K_ci_* = 0.012 and *K_cd_* = 13.5.

### 5.2. Scenario I: Sensor Output Error Fault in Steady State Operating Conditions

The simulation scenario I assumes a fault with aliasing noise in the current sensor output signal in DG steady state operating conditions to verify the ability of fault-tolerant control to suppress sensor output error faults.

It is assumed that a normally distributed random noise signal with a variance of 1 is aliased into the output signal of the A-phase current sensor within the period of 1 s to 1.5 s. In the period of 2.5 to 3 s, a normally distributed random noise signal with a variance of 1 is aliased into the output signal of the A-phase voltage sensor. These faults result in sensor output errors in two period of 1 s to 1.5 s and 2.5 s to 3 s.

**Figure 4:**
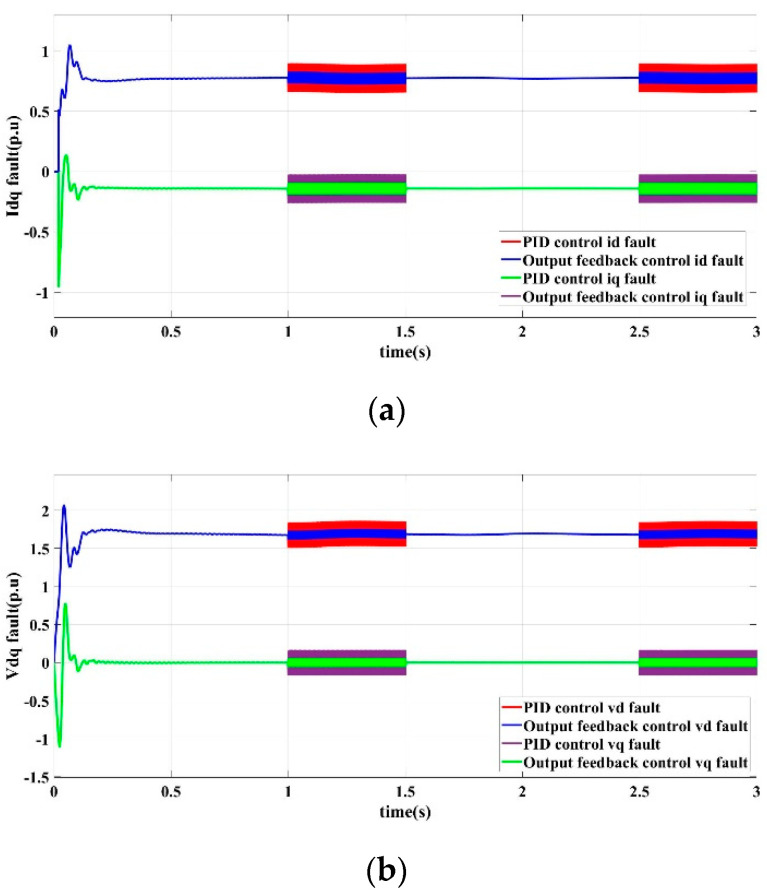
PID control and output feedback control response curves in scenario I. (**a**) *d*-axis and *q*-axis current output. (**b**) *d*-axis and *q*-axis voltage output.

In Scenario I fault conditions, PID control and output feedback control are applied, respectively. The output response curves of the DG in islanded microgrid are shown in Figure 4.

Figure 4a shows the output current response curves of the *d*-axis and *q*-axis in DG steady state operating conditions based on PID control and output feedback control. It can be seen that the currents are distorted in two period of 1 to 1.5 s and 2.5 to 3 s. The current fluctuation range based on output feedback control is between 0.25 pu and 0.4 pu. In contrast, the current fluctuation range based on PID control is between 0.55 pu and 0.9 pu. Figure 4b shows the output voltage response curves of the *d*-axis and *q*-axis based on PID control and output feedback control. Theoretically, the *d*-axis output voltage *v_d_* can reach a stable state and keep the output unchanged, while the *q*-axis output voltage *v_q_* tends to 0 under the control of the phase-locked loop mechanism. However, due to the faults of sensor output errors, the output voltage is distorted in two period of 1 s to 1.5 s and 2.5 s to 3 s, and the fluctuation range based on output feedback control is between 0.15 pu and 0.25 pu, while the fluctuation range based on PID control is between 0.5 pu and 0.85 pu. It can be seen that when the sensor has an output error fault, the suppressed performance of the output feedback controller is better than that of the PID controller.

**Figure 5:**
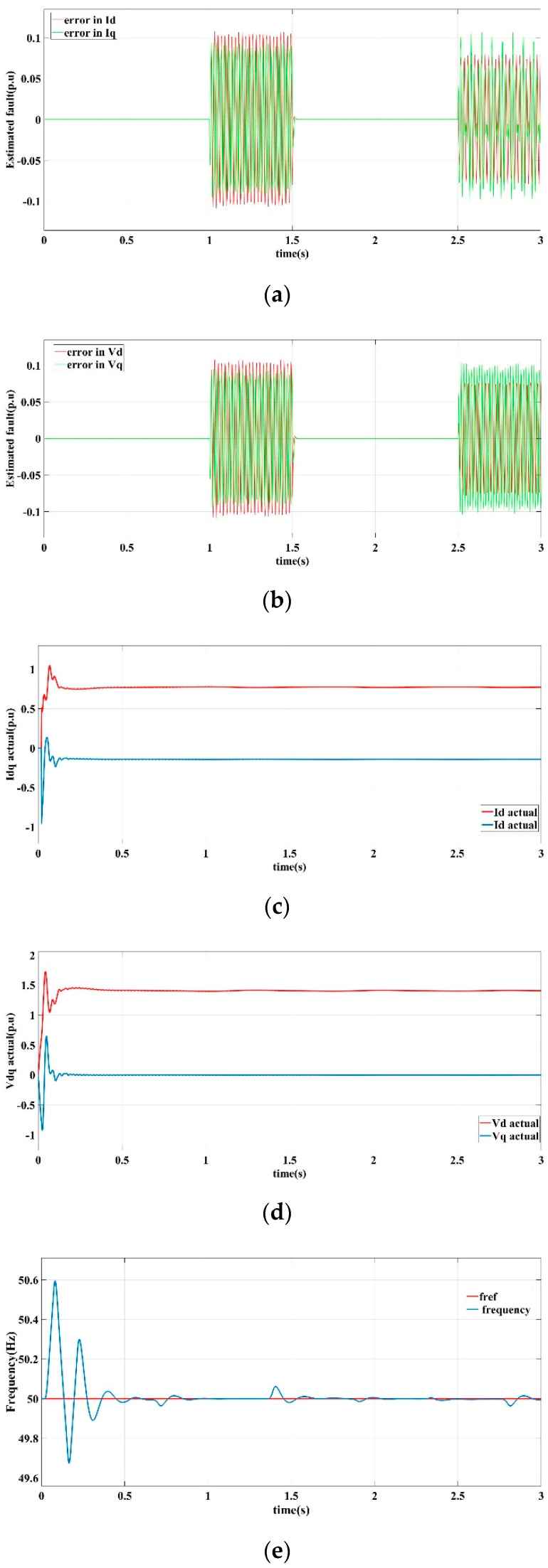

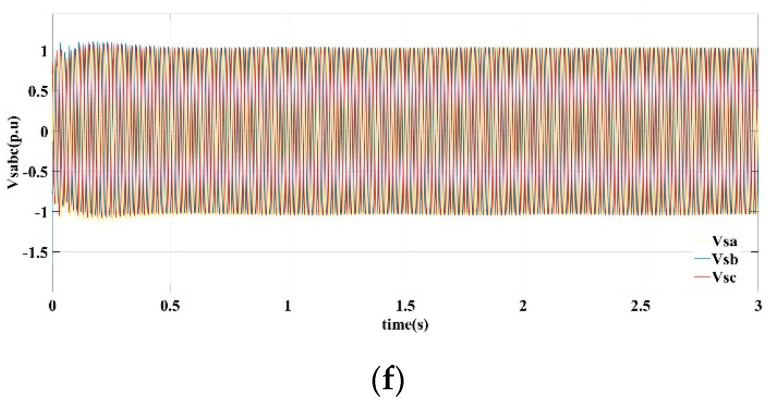
Fault-tolerant control response curves in scenario I. (**a**) Current error estimation of *d*-axis and *q*-axis, (**b**) Voltage error estimation of *d*-axis and *q*-axis, (**c**) *d*-axis and *q*-axis current output, (**d**) *d*-axis and *q*-axis voltage output, (**e**) Frequency output, (**f**) Three-phase voltage output.

Figure 5 shows the current and voltage output response curves of the DG in the islanded microgrid in scenario I operating conditions by the proposed fault-tolerant controller.

Figure 5a is the learning observer estimation curve of the detection current error of the *d*-axis and the *q*-axis. Figure 5b is the learning observer estimation curve of the measured voltage error of the *d*-axis and the *q*-axis. Figure 5c shows the output current response curve of the *d*-axis and *q*-axis. Figure 5d is the output voltage response curve of *d*-axis and *q*-axis. Figure 5e is the frequency response curve. Figure 5f shows the three-phase voltage output of the DG.

It can be seen that the learning observer obtains an accurate and quick estimate of the sensor error. Through fault compensation, the output current is relatively smooth, and the *d*-axis output voltage *v_d_* reaches a stable state and remains constant, while the *q*-axis output voltage *v_q_* tends to 0 under the control of the phase-locked loop mechanism. The frequency is basically stable at 50 HZ, and the fluctuation does not exceed ±0.1 HZ. In summary, the proposed fault-tolerant control strategy has a good ability to suppress sensor output error faults to ensure the stability and robustness of the microgrid bus voltage, as well as improve the steady state operating performance of the DG in an islanded microgrid.

### 5.3. Scenario II: Sensor Detection Precision Fault in Dynamic Power Operating Conditions

The simulation scenario II assumes that the sensor output signal has a detection precision fault, such as phase angle error or input/output ratio error, in dynamic power operating conditions. This simulation scenario is to verify the ability of the proposed fault-tolerant controller to suppress the sensor precision fault.

It is assumed that a 5% phase angle error is aliased into the current detection signal over a period of 1 s to 1.5 s. A 5% phase angle error is aliased into the voltage detection signal over a period of 2.5 s to 3 s. These faults result in sensor output precision fault in two period of 1 s to 1.5 s and 2.5 s to 3 s.

**Figure 6:**
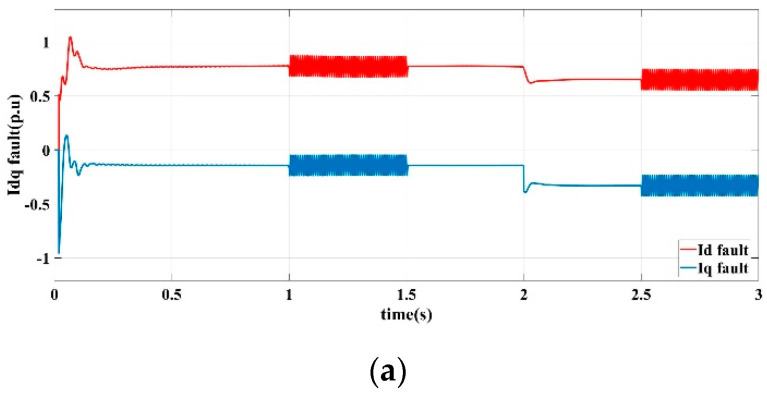

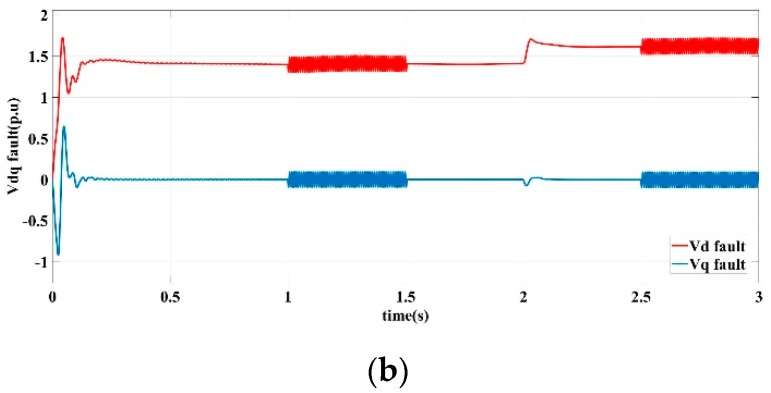
Output feedback control response curves in Scenario Ⅱ, (**a**) *d*-axis and *q*-axis current output, (**b**) *d*-axis and *q*-axis voltage output.

In the scenario II fault conditions, when only controlled by the output feedback method without a fault-tolerant control mechanism, the output response curves of the DG in the islanded microgrid are shown in Figure 6.

Figure 6 shows the current and voltage output response curves of the *d*-axis and *q*-axis in dynamic power operating conditions. It can be seen that the current and voltage are both distorted and cannot stabilize in two periods of 1 s to 1.5 s and 2.5 s to 3 s. Due to the sensor detection precision fault and the lack of fault-tolerant control, the current fluctuation range is between 0.1 pu and 0.25 pu, while the voltage fluctuation range is between 0.2 pu and 0.3 pu.

**Figure 7:**
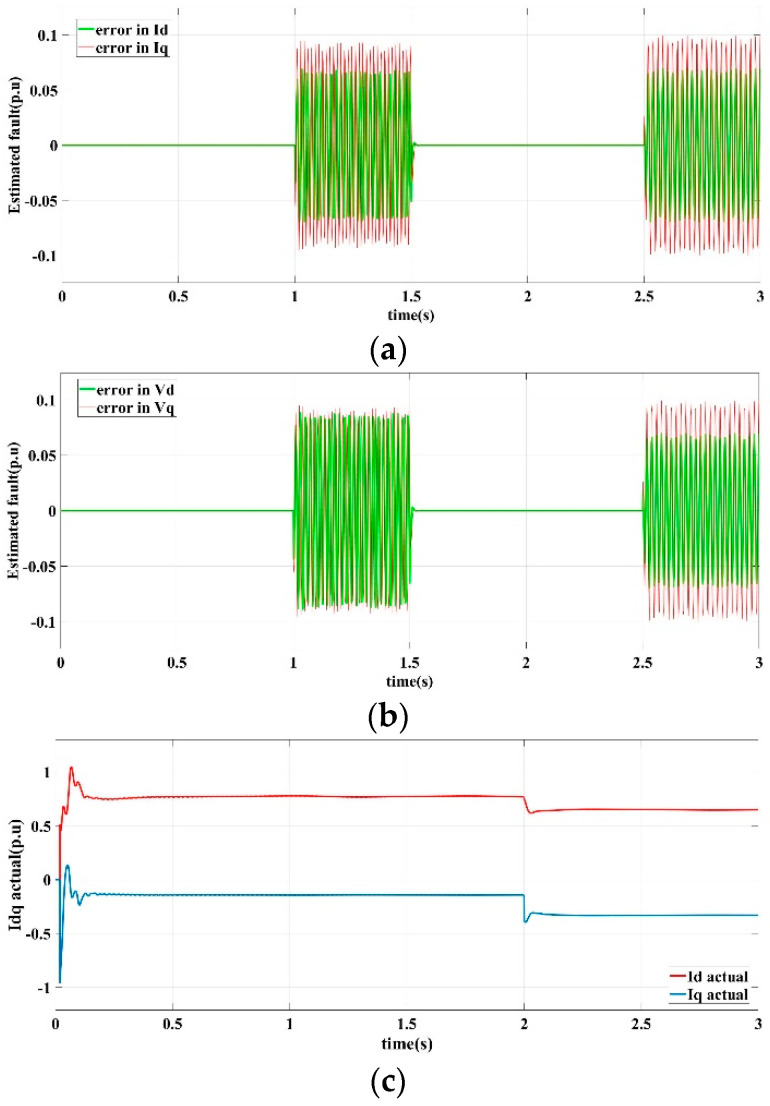

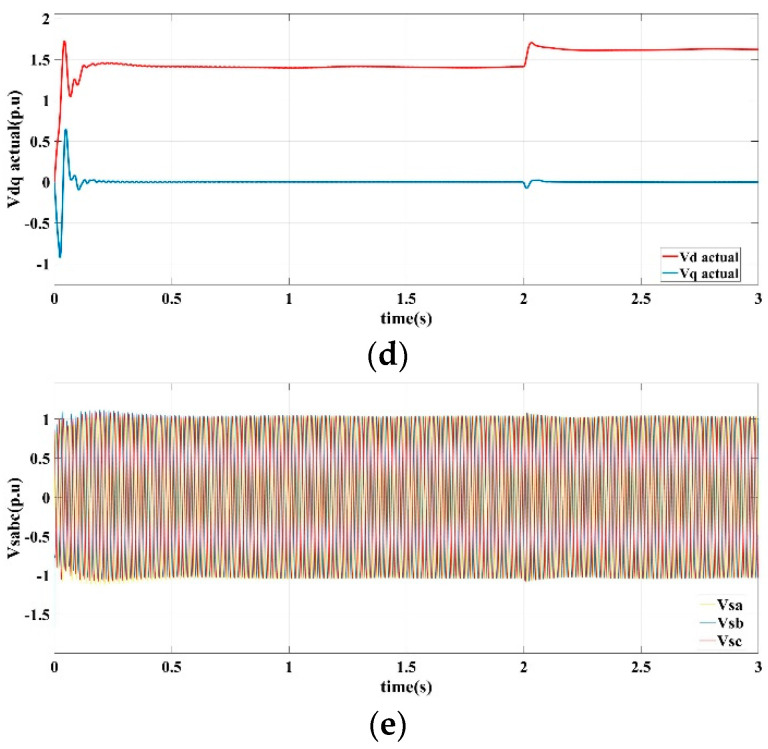
Fault-tolerant control response curve in scenario II. (**a**) current error estimation of *d*-axis and *q*-axis (**b**) voltage error estimation of *d*-axis and *q*-axis (**c**) *d*-axis and *q*-axis current output, (**d**) *d*-axis and *q*-axis voltage output, (**e**) Three-phase voltage output.

Figure 7 shows the current and voltage output response curves of the DG in the islanded microgrid in scenario II operating conditions by the proposed fault-tolerant controller.

Figure 7a demonstrates the learning observer estimation curves of the detection current error of the *d*-axis and the *q*-axis. Figure 7a shows the learning observer estimation curves of the detection voltage error of the *d*-axis and the *q*-axis. Figure 7c shows the output current response curves of the *d*-axis and *q*-axis. Figure 7d shows the output voltage response curves of *d*-axis and *q*-axis. Figure 7e shows the three-phase voltage output of the microgrid.

It can be seen that the learning observer realizes an accurately and quickly estimate of the sensor precision fault. The current and voltage curves are relatively smooth and stable. In summary, the proposed fault-tolerant control strategy has a good ability to suppress sensor precision fault to ensure the stability and robustness of the microgrid bus voltage, as well as improves the operating performance of the DG in the islanded microgrid in the condition of dynamic power operation.

### 5.4. Scenario III: Sensor Signal Lose and Transient Single-Phase-to-Ground Short-Circuit Accident Conditions

The simulation scenario III assumes that a single-phase-to-ground short-circuit accident occurs in the A-phase of the microgrid during the period of 2 s to 2.5 s. In addition, the A-phase current sensor detection signal is lost in the period of 1 s to 1.5 s, and the A-phase voltage sensor detection signal is lost in the period of 2.5 s to 3 s.

This scenario III is to verify the ability of the proposed fault-tolerant controller to suppress the sensor signal loss fault in the complex single-phase-to-ground short-circuit accident conditions.

**Figure 8:**
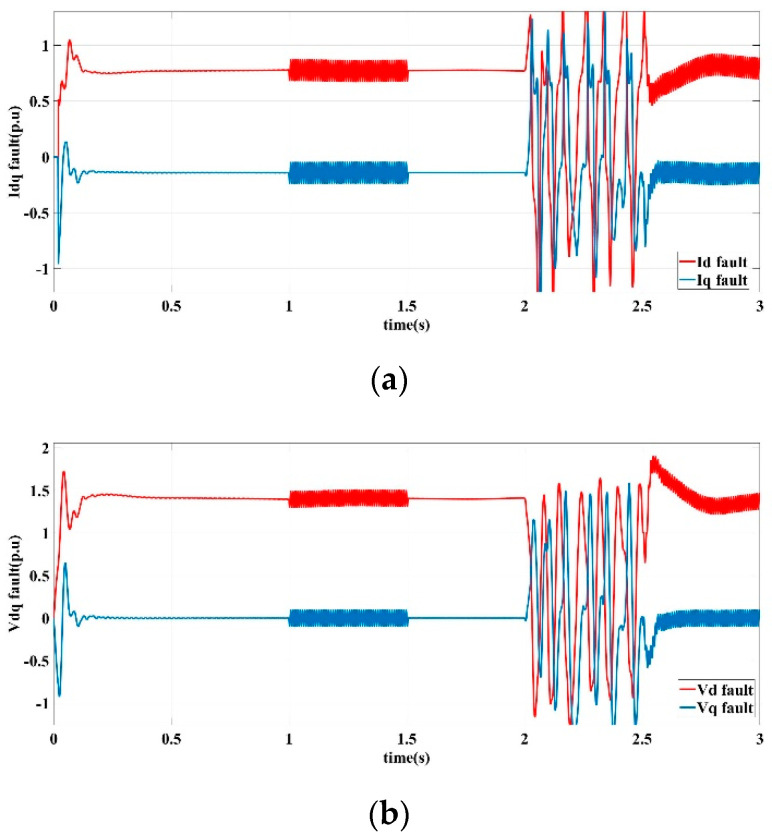
Output feedback control response curves in scenario Ⅲ. (**a**) *d*-axis and *q*-axis current output, (**b**) *d*-axis and *q*-axis voltage output.

In Scenario III fault conditions, when only controlled by output feedback method without fault-tolerant control mechanism, the output response curves of the DG in the islanded microgrid are shown in Figure 8.

Figure 8 shows the current and voltage output response curves of the *d*-axis and *q*-axis in sensor signal loss operating conditions. It can be seen that the current and voltage are distorted in two period of 1 s to 1.5 s and 2.5 s to 3 s. The current fluctuation range is between 0.25 pu and 0.5 pu, while the voltage fluctuation range is between 0.2 pu and 0.65 pu. Figure 8 illustrates that the microgrid cannot operate stably under faulty conditions.

**Figure 9:**
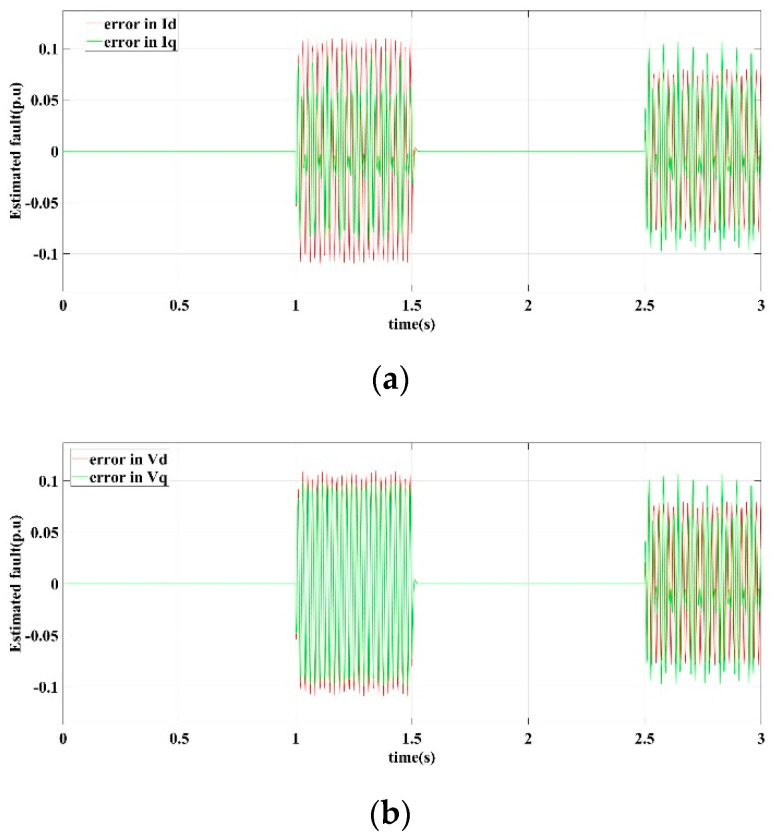

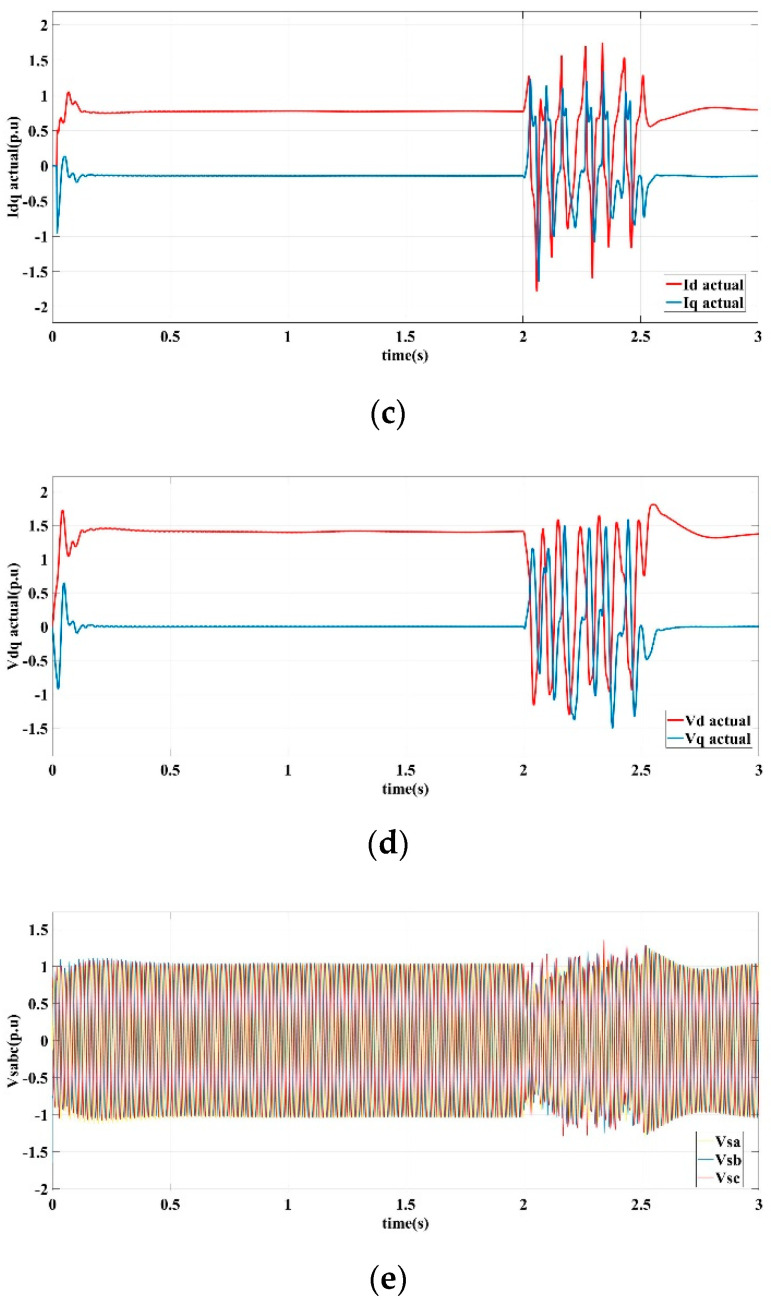
Fault-tolerant control response curves in scenario III. (**a**) Current error estimation of *d*-axis and *q*-axis, (**b**) Voltage error estimation of *d*-axis and *q*-axis, (**c**) *d*-axis and *q*-axis current output, (**d**) *d*-axis and *q*-axis voltage output, (**e**) Three-phase voltage output.

Figure 9 shows the current and voltage output response curves of the DG in the islanded microgrid in scenario III operating conditions by the proposed fault-tolerant controller.

Figure 9a shows the learning observer estimation curves of the detection current error of the *d*-axis and the *q*-axis. Figure 9b demonstrates the learning observer estimation curves of the detection voltage error of the *d*-axis and the *q*-axis. Figure 9c shows the output current response curves of the *d*-axis and *q*-axis. Figure 9d shows the output voltage response curves of *d*-axis and *q*-axis. Figure 9e shows the three-phase voltage output of the microgrid.

It can be seen that under the conditions of a single-phase-to-ground short-circuit accident, the proposed fault-tolerant control strategy also has a good ability to suppress the loss of sensor signals. Before and after the accident occurs, even if there has been a sensor signal loss fault, it can still maintain sensor fault tolerance, and ensure the stability and robustness of the microgrid bus voltage.

## 6. Conclusions

Based on the analysis of the control system structure and operation control method of the DG in the islanded microgrid, this paper aimed at examining the robust control problem of the system with sensor faults and uncertain disturbances. Firstly, a *dq* coordinate system model considering sensor faults was established; secondly, fault tolerant control structure based on fault output signal conversion filter and PD-type learning observer was constructed to realize online estimation and the real-time compensation of the fault signal; thirdly, a fault tolerant control strategy combining the output feedback control and learning observer was proposed to compensate an unexpected sensor fault. Finally, the simulation of multi-scenario sensor fault schemes were carried, and the experimental results verify that the proposed fault-tolerant control strategy has strong sensor fault tolerance and adaptability, as well as the high ability to enhance bus voltage stability and the robustness of the microgrid.

## Figures and Tables

**Table 1:** DG circuit parameters.

Parameter Name	Numerical Value
DC side voltage *V_d_*	800 V
Inductance *L*	10 mH
Resistance *R*	3 mΩ
Capacitance *C_f_*	500 μF
Inverter switching Frequency *f_sw_*	12 kHz
Frequency	50 Hz

## Data Availability

The data used to support the findings of this study are available from the corresponding author upon request.

## Nomenclature

*v_abc_*(*t*)	inverter output voltage
*i_abc_*(*t*)	inverter output current
*v_sabc_*(*t*)	DG voltage
*i_Labc_*(*t*)	DG current
*m_abc_*(*t*)	PWM modulation signal
*m_d_*, *m_q_*	modulation signal in *dq* coordinate
*i_d_*, *i_q_*	inverter *dq* coordinate current
*v_d_*, *v_q_*	inverter *dq* coordinate voltage
*v_sd_*, *v_sq_*	DG *dq* coordinate voltage
*i_Ld_*, *i_Lq_*	DG *dq* coordinate current
*ω*	angular frequency
*f*	frequency
*L*	inductance of the *LC* filter
*C_f_*	capacitance of the *LC* filter
*θ*	phase angle of the inverter
*R*	total equivalent loss resistance of the inverter
*x*	state variable
*z*	disturbance term
*y*	DG output variable with sensor fault
*y_s_*	DG output variable with normal sensor
*v_ref_*	reference input voltage vector
*K_p_*, *K_i_*	proportional gain matrix and integral gain matrix
*f_s_*	fault signal of the DG output current and voltage
*f_i_*	current detection error
*f_v_*	voltage detection error
*T*, *Q*	learning observer matrix
